# Why do patients struggle with their medicines?—A phenomenological hermeneutical study of how patients experience medicines in their everyday lives

**DOI:** 10.1371/journal.pone.0255478

**Published:** 2021-08-06

**Authors:** Joanne M. Fuller, Emmelie Barenfeld, Inger Ekman

**Affiliations:** 1 Institute of Health and Care Sciences, Sahlgrenska Academy, University of Gothenburg, Gothenburg, Sweden; 2 Gothenburg Centre for Person-Centred Care (GPCC), Sahlgrenska Academy, University of Gothenburg, Gothenburg, Sweden; 3 Department of Occupational Therapy and Physiotherapy, The Sahlgrenska University Hospital, Gothenburg, Sweden; Maastricht University Medical Center, NETHERLANDS

## Abstract

Why do so many people struggle with their medicines despite decades of research on medicines taking? Research into how people experience medicines in their everyday life remains scarce with the majority of research in this area of focusing on whether or not people take their medicines as prescribed. Hence, this study used a phenomenological hermeneutical qualitative design to gain a deeper understanding of individuals’ perspectives on the lived experience of medicine-taking. Findings from this study highlight five main themes where participants experience medicines as: 1) life-saving and indispensable, 2) normal and a daily routine, 3) confusing and concerning, 4) unsuitable without adjustment, and 5) intrusive and unwelcome. These results can be the basis for mutually agreed prescribing through a co-creative approach that aims at enhancing open and honest dialogues between patients and healthcare professionals in partnership about medicines.

## Introduction

Patients are commonly prescribed numerous medicines when they interact with our healthcare system which continues to rely heavily on the use of medicines [1, 2]. Yet this increased number of medicines is not without risk nor negative effects on the recipient’s everyday life. It is well-documented that polypharmacy, defined as four or more concurrent prescribed medicines, increases the risk for adverse drug events (ADEs) [3, 4]. Whilst the primary aim of prescribing medicines is to improve health and quality of life, the reverse is oftentimes experienced due to the use of medicines leading to a significantly increased number of avoidable hospital admissions and preventable deaths [3, 4]. The purpose of prescribing medicines to patients with chronic conditions, for example, common conditions such as chronic heart failure (CHF) and chronic obstructive pulmonary disease (COPD), is to significantly reduce both morbidity and mortality [5, 6]. However, for such benefits to occur patients need to understand why their medicines have been prescribed and choose to take their medicines as directed. As many as 50% of patients struggle with taking their medicine as prescribed [7, 8], which leads to unnecessary individual and societal costs [6]. This problem has been researched for decades where the focus has been on patients’ medicine-taking behaviour [9, 10] as the cause and area for improvement [11].

Several innovative interventions that aim to improve medicine-taking (often referred to as adherence [12]) have been the result of research into barriers to taking medicines as prescribed [10, 13]. Factors that are known to influence how patients take medicines include risk of side effects, patient direct cost, time imposition, interruption to one’s daily routine and more [10]. Many promising interventions have reduced the proportion of patients not taking their medicines precisely as directed [13] where individual level interventions have aimed to have a patient focus to both informatively enable or help the patient in their action of taking their medicines. However, oftentimes the positive results are not retained over a longer period (at least six months) and hence, these positive benefits are yet to change the overall landscape of medicine-taking [14]. Therefore importantly, decades later we still have similar percentages of patients who do not receive the information or support they need to take their medicines as directed [14]. Furthermore, such interventions into medicine-taking often do not take into account factors other than clinical aspects [14]. For instance, a patient’s usage of medicines does not occur in isolation and may be influenced over time by factors other than their individual experiences of medicines. Hence, sociological factors that have been changing particularly over recent decades, such as, the “pharmaceuticalization of society” which includes driving forces such as consumerism, regulatory policies, pharmaceutical industries’ marketing and biomedicalization also influences patients in their medicines usage [15].

Research on patients’ experiences of taking their medicines has identified medicine-taking as a complex social interaction [9, 11]. Medicine-taking involves many factors including both the medicines and patients themselves, as well as family members, friends, and clinicians which all influence use of medicines. This complex social interaction is highlighting the need for clinicians to be aware of their own role, how their communication and lack of acknowledgement of patients’ previous experiences and context may influence how patients construct their thoughts around medicines [9, 16, 17] rather than basing their practice around the recommendations of guidelines alone. Furthermore, guidelines are written to inform treatment decisions, excluding the lived burden of medicines as perceived and experienced by the patients [18]. Yet it is the lived experience that is both vital and often the decisive factor as to whether or not patients take medicines as prescribed [19]. Moreover, the *Medicines Optimisation* guideline released in 2015 by the *National Institute for Health and Social Care Excellence* (NICE) states that medicines optimisation is applicable “to people who may or may not take their medicines effectively” [20]. These guidelines are intended to improve medicine-taking in the United Kingdom by guiding “the safe and effective use of medicines to enable the best possible outcomes” also raise how vital it is “to ensure a person is taking their medicines as intended and can support the management of long-term conditions, multimorbidities and polypharmacy [20].

Published research on patients with common chronic conditions and their lived experience of medicine-taking does exist is in the biomedical sphere [21] yet remains scarce [22] as opposed to in the sociological domain [11, 17]. Additionally, some research on medicine-taking more often reports on those patients who do take their medicines as prescribed in contrast to those patients who do not take their medicines as prescribed [23]. To understand patients’ experiences about how medicines impact on their everyday life and how patients perceive the medicines prescribed to them it is therefore vital to understand factors influencing how patients decide to take their medicines or not.

Hence, the aim of this study is to gain an in-depth understanding of how patients experience their medicines in their everyday life.

## Method

### Ethics

All included participants received oral study information and provided their signed informed consent. The study was approved by the Regional Ethical Review Board in Gothenburg (DNo. 063–17) and conforms to the principles outlined in the Declaration of Helsinki.

### Design

An exploratory qualitative interview study, the data were analysed using phenomenological hermeneutics [24]. This approach is designed to develop deeper understanding of individuals’ perspectives by interpreting their narratives as texts and is suitable to uncover the reality of medicine-taking by moving beyond descriptive levels of data [25, 26].

### Setting and participants

The sample was a convenience sample of patients who were expected to provide rich data [27] around medicine-taking and who were registered at primary care centres in Western Sweden and participating in a larger randomised controlled study (RCT) [28]. Healthcare professionals identified patients through screening medical records guided by pre-defined sampling criteria chosen to identify those patients with experience of medicine-taking [27]. These criteria were adult patients who had participated and completed the final data collection in the RCT, who were primarily diagnosed with CHF or COPD and other secondary chronic illnesses, and who also had personal experience of taking any prescribed medicines. After consent, the first author contacted the patients by telephone to schedule an appointment for an interview. Participants and the interviewer were unknown to each other prior to this contact. A total of 14 participants were asked to participate of which 11 agreed to participate. Reasons for declining participation (n = 3) were no interest in participating and inability due to hospitalisation.

### Data collection

All interviews were conducted by the first author, a female research pharmacist with a PhD, who had several years’ experience in community pharmacy practice and was trained in qualitative research with some experience from a previous qualitative study [29]. Participants were informed that the interviewer was a researcher with an interest in medicine-taking from the patient perspective. The interviewer’s background as a pharmacist was only disclosed to those participants who inquired directly about the interviewer’s background. Data collection occurred over a five-month period from November 2018 to March 2019, allowing the interviewer time for reflection after each interview.

All interviews were face-to-face with the majority conducted in the participants’ homes to encourage an ‘at-ease’ sense in the participants, making it easier to talk about their feelings and experiences. Two participants opted to be interviewed at the University or a café at their convenience.

The unstructured interviews were started with a single open-ended question. A pilot interview was performed to test an interview guide used only as a support when patients needed encouragement to talk more. The pilot interview was not included in the analysis. The support interview guide included probes to stimulate further conversation such as, how participants felt generally about medicines. These probes were formulated as dot-points and not as complete questions. Participants were first asked to describe their thoughts about medicines (as a response to the opening question “what do you think of when you think about your medicines?”). Participant responses were guided to include descriptions and examples of medicines in their everyday life with the help of follow-up questions to the initial question. For instance, if the interviewee had mentioned something that the interviewer felt could be developed more, a follow-up question such as “that’s interesting—can you tell me more about that?” was asked. A pilot interview was performed to test the interview guide and resulted in no changes to the guide. The pilot interview was not included in the analysis. The guide included probes to stimulate further conversation such as, how participants felt generally about medicines. These probes were formulated as dot-points and not as complete questions. Field notes on body language and other relevant information such location and environment for the interview were taken to help with the data analysis. All interviews were audio-recorded and transcribed verbatim.

### Data analysis

To reach the participants’ deeper perceptions of their medicines, the phenomenological hermeneutical methodology as developed by Lindseth and Norberg was used to analyse the transcripts [24]. Underpinning this methodology is Ricoeur’s theory of interpretation where meanings of lived experience can be understood through narrative interpretation of texts (in this case, interview transcripts and the observational notes made in the interview) [30].

This method has been used in numerous studies e.g. [31, 32] and can be described in three interrelated steps:

- Repeated reading of the text in order to acquire a naïve understanding of the content.
- Structural analysis when the text is divided into meaning units that are condensed and form the themes.
- Finally, the naïve understanding and the themes are weighed together to create a comprehensive understanding of the studied phenomenon.

In contrast to a linear analytic process, the hermeneutic circle is used throughout this phenomenological hermeneutical analysis [33]. There is an ongoing circular interpretation of each part or meaning unit with the naïve understanding of the whole text in order to understand the whole text and to enable the researcher to understand the part and vice versa [33]. Each interview transcript was read through several times to form a naïve understanding [24]. The lead researcher engaged in self-reflexivity [34] in order to critically examine their own identity and research approach to reduce potential bias and influence during the naïve reading [35]. Next, a structural analysis was conducted to examine each interview transcript text as a whole and identify line-by-line meaning units that related to both the study aim and naïve understanding. These meaning units were then condensed into sub-themes, compared and grouped into themes that captured the participants’ perception of medicines in their everyday life. Using the hermeneutic circle concept [33], several analyses guided by the naïve understanding compared the text as a whole with the meaning units and themes. Finally, based on the author’s preunderstanding, naïve reading and structural analyses a comprehensive interpretation was made (JF, EB, IE).

## Results

### Participants

General characteristics for the group are shown in Table 1. The mean length of interviews was 36 (range 15 to 71) minutes and resulted in 357 pages of transcribed text in total.

**Table 1:** Participant characteristics (n = 11).

**Sex**	Female	3
	Male	8
**Age (years)**	Mean	70.9
	Median (range)	73 (53–85)
**Civil Status**	Living alone	4
	Partner	7
**Currently Working**	Yes	1
	No	10
**Education Level completed**	Primary School	1
	Secondary School	3
	Technical College or University	7
**Diagnosis**	COPD	7
	CHF	4
Number* **of Medicines**	Mean	4.4
	Median (range)	4 (1–7)

General characteristics for the group.

*Prescribed medicines listed in patient medical journal accessed post interview for group description purposes only.

### The naïve reading

Overall, the participants could be grouped into two general groups where one group’s medicines were generally unproblematic and fit well into their daily lives. The medicines had a clearly defined place in their lives as life necessary due to their positive and tangible life-saving results.

In contrast, a second group was more negative about medicines in general and identified several problems with medicines in their daily lives, including frustration and uncertainty with their medicines and concerns about the medicines’ purpose and overall usage.

### Structural analyses

Five themes relating to the how the participants experienced their medicines in their everyday life were identified in the structural analyses and are as follows:

- Life-saving and indispensable
- Normal and a daily routine
- Confusing and concerning
- Unsuitable without adjustment
- Intrusive and unwelcome

A table showing an example of the structural analysis process will be provided for the first theme.

Each theme is described in detail below with supporting quotations from the interviews presented in italics with the participant number (1–11) and sex shown in brackets (Female = F; Male = M).

#### Lifesaving and indispensable in the patient’s life

The participants described how medicines were accepted as well-suited and a necessity in their daily life. Participants’ acceptance also included reluctance to experiment in *not taking* their medicines and how they dare not take such a risk due to the probable life-threatening results. participants also described feeling good or much better in daily life due to their current medicines particularly when compared with living with the illness previously untreated and the consequent symptoms. The awareness of the disease’s seriousness and the reality that the medicines’ mechanisms kept one alive was regularly discussed. For example, one participant stated that:

“*If I don’t take*, *[or I] am careless*, *or mismanage my medicines*, *then it’s my life at stake*. *If I stop taking my medicines*, *it would be a slow suicide because my heart would fail*. *Absolutely*. *That’s how it is*.”

(P11; M).

Table 2 shows a condensed view of the structural analysis process for the ‘*Lifesaving and indispensable’* theme, that is, how the text from relevant meaning units were condensed and then fit within the overarching theme.

**Table 2:** Structural analysis process.

Meaning units	Condensation	Theme
It’s negative that I have been sick. I would rather be well. … Yes, now that I am [sick] I am grateful that [the medicines] exist. (P5; M)	I am grateful for these medicines that keep me alive	Lifesaving and indispensable
I realize that medicines impact your health, and you have to take it for it to work when your body has failed you as has my heart…. Conversion is talked about, but it was never considered in my case. It’s just medicines that applies in my case. (P7; M)	Medicines can make up for where my body has failed me	
Yes, I understand that I must have [my medicines], …if I didn’t have them, then I don’t know if I would be sitting here today. (P9; M)	I may not be alive today if I hadn’t had my medicines	
Medicines are good—what else can I do? If I don’t take medicine for my high blood pressure, then I could have another stroke. (P10; M)	My medicines protect me from a stroke	
Without my medicines I don’t have the stamina to work, or even walk to the bus, or anything. (P11; M)	I function normally because of my medicines	

Structural Analysis Process for the Theme, *Life-saving and indispensable*.

#### Normal and a daily routine in the patient’s life

Participants described taking medicines as a normal part of their daily routine and at times habitual. They also talked about medicines as being a regular and normal part of their everyday life as shown in the following quote:

“*It*, *it is a part of my morning routine*. *So*, *it’s… And I have*, *I have two of those—I fill two of those [weekly] dosage aids [dosettes]*. *Yes*, *yes*, yes…so that I don’t have to sit and fiddle with all the containers *laugh* … I will fill two [dosettes] today, *as it’s time now… I had calculated it so that it would be enough until today*”

(P2; M).

Participants described how their medicines were a continual part of their life to be taken every day for the rest of their life, how they had a system that made taking their tablets easier and less time consuming, and that this daily task was not a problem for them at all. Medicines and planning ahead as a normal factor of life was also talked about. Being embedded in their daily routine meant that the action of medicines taking added no cognitive burden to their everyday life. This daily routine action was strengthened by using a dosage aid which was common for several participants. For some participants, family members were involved in filling these dosage aids or monitoring prescriptions and medicine supply to ensure that there were enough doses available to fill the dosage aid/s. This was talked about in terms of planning ahead as one does when planning daily life in general and naturally. Additionally, this was relevant in other life situations, for instance, when one participant had been on holiday, he described how ensuring that he had enough medicines with him to cover his trip as a normal part of the planning and packing for the holiday. Moreover, some participants talked about how it didn’t matter if a single dose meant taking one or more tablets as long as one was taking a dose anyway, that is, that it was normal routine to take medicines so an additional tablet to swallow per dose did not add any burden. Additionally, one participant linked his daily routine of taking his medicines to the routine of giving his pet daily medicine as highlighted in the following quote:

“*Yeah and now*, *I have [the pet] to think about too*, *as in a slight constraint that he has to have [his medicine too] so*, *so far it’s gone well… it’s rare that I forget it…*”

*(P9; M)*.

#### Confusing and concerning in the patient’s life

Some participants described how medicines in their everyday life also related strongly to a sense of confusion and concerns including lack of clarity as to why these medicines were prescribed for them. To some participants raising the subject of medicines led to them saying how they were confused about the actual purpose of the medicines. It had not been clearly communicated to them why they should take the medicines, or if the treatment was to be ongoing or not. Rather than provide a sense of security or assurance, the presence of the medicines in their lives lead to more questions rather than answers. It was also common for participants to be unsure about the medicines that they were taking in terms of alternatives, that is, not knowing if there were “better medicines” or “stronger medicines” that would be more beneficial and suitable to them. One participant stated the following:

“*Now I have this*, *[medicine name] it’s called*, *[medicine’s strength]*. *Is there a stronger dose of it*, *that I can get…*?”

*(P1; F)*.

The same participant continued to wonder as follows:

“*I don’t know if I should be honest because I don’t know what to expect … Is there a better alternative*? *That’s what you don’t know*. *Is there something that is better*? *And although I don’t know*, *this feels…it’s very hard to know just how* ***much*** *better I could be*? *But how should…yes*, *so that’s why it’s hard to judge if this will make it much better or if it doesn’t help at all or something like that*”

*(P1; F)*.

The participants had many unanswered questions about their medicines and the intended effects that were discussed around experienced side effects. At times, side effects were perceived as worse than the underlying disease being treated. Similarly, the concern that medicines may lead to other unwanted effects and concerns that the prescribing doctor could be relied upon to be aware of all such risks. Additionally, participants also experienced that a prescribed medicine had not given the expected result as stated in the following quote:

“*No*, *the doctor didn’t explain to me why the medicine hadn’t helped*. *She said that sometimes it’s hereditary*. *Sometimes it comes from the food*, *what you eat*. *But she doesn’t know why [the medicine] didn’t help*. *[She said that] it should help*. *It usually helps …*”

*(P8; M)*.

Similarly, another participant wondered if the medicine gave any effect at all as stated in the following quote:

“*I don’t feel like the medicines do any good*. *But maybe they do*”

*(P4; F)*.

Questions about the possibility of interactions between medicines also falls under the theme *confusing and concerning in the patient’s life*. Participants reported that they often felt there was not enough time to raise this common concern during appointments. Instead, they described how prescriptions were simply renewed with limited discussion, and how their concerns or experiences regarding interactions were not acknowledged during this process. Some participants also discussed their concern when not being able to meet with their doctor to discuss their medicines and how they often were given appointments with a specialist nurse rather than with their doctor despite several explicit requests, indicating that their wishes had not been respected.

Concern of being misunderstood when communicating about medicines with clinicians was also discussed by a participant. Although this participant had a clear understanding of their medicines and regularly used these medicines as directed, they described in detail an event where they had previously expressed uncertainty about a medicine. The prescribing doctor misunderstood what the participant had said which led to an entry about in the medical record. Following this entry, the participant had an ongoing feeling of misjudgement in following encounters with other clinicians, as described in the following quote:

“*The note remained*. *It stuck around for a long time*. *And that’s a bit scary*, *a simple misunderstanding… although I had said to remove it*, *it’s a misunderstanding I said*.*” … “And you think*, *I can*, *I can speak for myself*, *… but older*, *weaker people*, *in such cases*, *how wrong can it go … that upset me … how long that misunderstanding stuck around*”

*(P11; M)*.

This participant went on to then express concern as to how they had been perceived as a patient *“they got the wrong image of me as a patient … one who does not believe in the medicine’s function and benefits … and that’s not me at all*.*” (P11; M)*.

#### Unsuitable without adjustment in the patient’s life

Medicines were described as being made to fit into the participants’ lives through changes made after a process of adaption. These changes were in agreement between the participants and their healthcare professionals. There were two major types of changes for medicines to suit in one’s life; 1) lifestyle adaption to accommodate medicines and 2) adjustment of the medicines to suit the patient. Lifestyle adaption included having some lifestyle restrictions and a longing for one’s previous life without medicines as exemplified by the following quote where the adaption of being free to consume alcohol had due to the risk of alcohol possibly interacting with a medicine taken by the patient. This adaption was perceived by the participant in both a positive and negative light:

“*… like when we were away on holiday you know*, *it was hard*, *well not hard as such*, *when you wanted to have a drink in the evening and so on… But you can drink non-alcoholic drinks as well*. *It’s just a bad habit to drink alcohol [spirits]*”

(P5; M).

Several participants disclosed how their medicines had either been changed to suit them or the dose adjusted to attain the wanted effect after discussion with the prescribing doctor, or in contrast to the confusing and concerning theme where a reciprocal conversation had not occurred. This process of adjusting the medicine or dose may have taken some time rather than being immediate as described in the following quote:

“*I had high blood pressure…and my general practitioner said that we would have to [test anti-hypertensive medicines] …it took*, *it took…it hung in there for about a…a test period of three months*. *… so*, *because of that I had a low pulse and…you can’t test all sorts of medicines*. *Um*, *so it took over a year before we got it right…*”

(P2; M).

Participants also spoke about being able to, in agreement with their clinicians, adjust the dose and timing of their diuretic medicines to suit their physical needs, for example when they observed that they were retaining more fluid than usual or had weighed significantly more than usual. This ability to adjust the medicine to suit was described in a positive light as their wishes had been recognised through a reciprocal conversation and allowed the participant to have a sense of control over both the illness and the medicine as shown in the following statement:

” *… so*, *so then she [the nurse] said that I could take two [diuretic tablets] the next day instead [of taking one tablet as the day before] so that the fluid doesn’t accumulate…apparently*, *you can do that… so* ***that*** *helps*”

(P4; F).

#### Life-intrusive and unwelcome in the patient’s life

Medicines as life-intrusive and unwelcome into one’s life highlighted the difficulties participants found when they tried to incorporate their prescribed medicines into their daily lives. This theme encompassed several factors that made this integration either difficult or not possible. Factors such as a lack of trust for the medicine itself or general mistrust towards any medicine at all, disappointment due to the lack of a clear tangible improvement from the medicines being taken, a misalignment between the identity of having an illness whilst otherwise actively striving to be fit and healthy through regular exercise, or feeling worse instead of feeling better from the medicine as shown in the following quote:

“… *a while back*, *no*, I was so dizzy and I felt unsure when I went out so I took my walking sticks with me … so I stopped taking [the medicine] *laughter in voice*”

(P4; F).

This theme also included factors such as the mental load or burden of having medicines in one’s life through either having to be aware of the medicines, the monitoring of blood levels of medicines or even feeling guilt or disappointment when the patient had forgotten to take the medicine as displayed in the following quote:

“*Yes*, *it [disappointment with myself] for example these medicines*, *that are about blood pressure and blood fl… um blood flowing… Blood thinner*, *yes… and it’s*, *they are*, *it is important that one take’s them*”

(P8; M).

Experiencing or worrying about the unwelcome possibility of side effects also fell under this theme with several participants either directly commenting or alluding to their concerns about side effects meaning that the medicines did not suit them. One participant weighed the medicine’s expected effect for something that she didn’t feel against a clearly experienced side effect stating:

“*And that [medicine] for asthma*, *I take it when*, *as needed … so it’s not direct … and the other one that was cortisone*, *that one I am supposed to take morning and evening … but now I don’t feel any [need for it] … because when I stopped using it I wasn’t hoarse or anything but now when I’ve been taking it for a week*, *I am hoarse* ***again****…*”

(P4; F).

## Comprehensive interpretation and discussion

This study aimed to provide an in-depth understanding of how patients experience the medicines they take for common chronic conditions. Five overarching themes were identified that conceptualise the meanings participants give to medicines in their everyday life and highlight how medicines are experienced as both positive and negative that do or do not fit with the person and/or their life and subsequently influences the medicines’ usage. These meanings included both existential and practical aspects within the participants’ lives, some of which have been reported in earlier research albeit in separate studies or differing populations or settings [9]. Hence, the identified themes have not been previously identified within a Swedish population of patients with common chronic conditions.

Baxter *et al* conducted phenomenological hermeneutic research in the area of health (specifically meanings of thriving for persons in nursing home) [36] and discussed a person’s decision-making as a metaphorical door that can be both opened or closed to allow access to the ‘new world outside’ according to the teachings of Gaston Bachelard, a philosopher and phenomenologist [37]. In our research, the door that represents a person’s decision-making that either enables or blocks a person’s medicine-taking behaviour was clearly influenced by their understanding of the ‘need’ for the medicine. For instance, those participants who described medicines as *‘lifesaving and indispensable’* had a clearer view of the possibilities the regular usage of medicines offered them that then influenced their decision to take their medicines as prescribed. They experienced their medicines as opportunities to improve or maintain their current health meaning that the medicines enabled them to access a better life with improved quality-of-life and less symptoms of disease. They even talked about a life that was lengthened in comparison to a life without medicines and if the disease was untreated. In direct contrast is the theme *‘life-intrusive and unwelcome’*, where it was difficult or impossible for these participants to see the possibilities that the medicines could offer them [37].

The participants who saw their medicines as ‘*life-saving and indispensable’* also had clear *intentions* regarding their life-long medicines, which is discussed by Granger *et al* in the study that assessed medication adherence investigated through content analysis of structured interviews of inpatients diagnosed with heart failure in the United States [38] using the Meaning-Response theory [38, 39]. Granger *et al* found intention as a major construct that was a commitment to taking life-long medicines as part of their ongoing life [38] which is also evident in our research. This intention was also evident in participants who discussed ‘*medicines as normal and a daily routine’*, who took their medicines due to their understanding and acceptance as to why they were taking the medicines, where there was little or no ambiguity as to the purpose of these medicines and the importance of taking them regularly. Hence this acceptance of theoretical underpinning that had been clearly and well communicated to them by their clinician/s led to regular, daily thoughts and actions to include the medicines into their everyday life. This was in contrast to those participants who struggled with accepting their prescribed medicines or grappled with expectations that had not been met by the medicines as displayed in the theme *‘confusing and concerning’*. Notwithstanding a lack of positive tangible results, these participants had not theoretically accepted the medicines as purposeful to them [16] nor had their uncertainties or frustrations been acknowledged or addressed by their clinicians. A reciprocal conversation had not occurred which has been identified as problematic in earlier research [40, 41].

Patients must be able to freely discuss their experiences of medicines and any reservations they may have to medicines in a reciprocal conversation that is enabled through the provision of an open and non-judgemental space. To enable a reciprocal conversation, a safe space must be provided where patients can freely discuss without judgement or time constraints so that those who need to can raise their concerns to enable treatment decisions to be made in partnership, that is, *made with patients* rather than *for* patients. This conversation needs to recognise patients as capable partners who are encouraged and afforded sufficient latitude to share their experiences on medicines [42, 43]. This in turn should lead to a dosage regimen or prescription in the form of a mutually agreed and tailored plan [8] such as with the participants in this study who discussed meanings that fit within the adjustment and adaption theme. Many models of prescribing exist that aim to be more inclusive of patients in decision-making around their medical treatment, including concordance, tailored prescribing, shared decision-making, clinical empathy, and patient-centredness [14, 41, 44, 45]. However, these models still remain *medicocentric* [44] as opposed to the recommended person-centred medicines’ optimisation [20] where the person in the role of the patient through equal partnership and co-creation can make their decisions based on medicinal guidelines and personal experience.

A UK hermeneutic interpretive study [46] conducted in patients with difficult asthma using corticosteroids describes a patient’s strong knowledge of their medicines and the underlying disease as *Knowledge as power* that led to an enabling pathway that resulted in participants using their medicines as directed and giving them a sense of having the ability to gain control over the illness. Additionally, a Danish phenomenological study of young women taking medicines however showed that even participants who were generally negative to medicines were willing to take medicines if they understood the benefits of the medicines and that such knowledge impacts people’s behaviour [9, 47]. Hence, as the theme ‘*confusing and concerning’* in the present study highlights, it was unclear to the participants how their prescribed medicines could benefit them. A more appropriate communication with the prescriber in which the patient feels they are given mental space and adequate time to discuss their needs and concerns has been highlighted in earlier research [48] particularly as their priorities often differ to that of the prescriber [49]. Even if a patient understands how effective or useful a medicine could be for them, they may still decide not to take the medicine as they simply do not want it based on the other factors it brings with it, such as side effects or other ‘risks’ that outweigh the ‘need’ or ‘benefits’ as shown in the selected clinical research that has been presented to them at the point of prescribing [9]. Medication-taking is complex and multi-facetted as is living with the benefits and possible side-effects of multiple medicines that may in turn be further complicated by an aging body or multi-morbidity [4, 9]. The same patient may find it relatively easy to take one medicine as directed but struggle with another, meaning that the same patient can be seen as both adherent and non-adherent at the same time—and this adherence may be a result of the severity of the illness being treated or by the complexity of the medicine regimen. There is no ‘one size fits all’ response to medicine-taking and its complexity which must be taken into consideration when planning interventions aimed at improving medicine-taking [9], hence the lack of progress in terms of adherence rates over the recent decades [10–12]. However, as shown by this study, attempting to understand the patients’ perspectives in this complexity is a vital starting point for our understanding as healthcare providers. A person-centred approach that focuses on co-creation through partnership [50] in prescribing would address such issues and is further discussed within medicines optimisation according to the *National Institute for Health and Social Care Excellence* (NICE) *Medicines Optimisation* guidelines [20, 51]. The co-creation of the treatment plan (or prescription) would make it easier for the patient to take the medicine as mutually agreed upon.

The theme ‘*unsuitable without adjustment’* highlights how changes both ways may be necessary to make medicines suitable to patients’ lives. Change in either a patient’s behaviour to accommodate a new medicine or type or dose of medicines can be challenging and difficult [14]. Prescribing pharmaceutical medicines includes both initiating treatment and dose adjustment. This is a process that at times may be ‘hit and miss’ where the ‘miss’ can represent ineffective treatment, possible side effects or even unexpected adverse events that in turn may lead to the changing of either the prescribed medicine itself or the dosage as discussed by some of those interviewed in this study. It is also important to acknowledge that patients may be correct in deciding not to take medicines as medicines are often not safe nor effective [47, 48, 52]. Despite patients also being within their individual rights to decide over their own body, they are often ladened with a ‘moral obligation’ to take medicines as prescribed regardless of the burden or risk these may involve [11]. *Hawking et al* state that this obligation is sustained through an ongoing “*morally laden discourse*” where the concept of non-adherence is “*depicted as a patient-related problem to be solved by the health sector*, *and as a [patient’s] personal failing”* [11]. This ‘problem’ of non-adherence *“invites moral assessments from [clinicians] regarding how patients use their medicines*” [11]. The ongoing medication adherence discourse plays to the traditional and hierarchal power imbalance between clinicians and patients and infers *“personal responsibility and blaming of individuals for ill-health*” [11].

The process of prescribing (perceived as morally good) typically involves the intention and expectation that medicines are both safe, effective and morally good which is in line with the *National Institute for Health and Social Care Excellence* (NICE) *Medicines Optimisation* guidelines which refers to the “safe and effective use of medicines to enable the best possible outcomes” [20, 51]. The NICE guidelines state that the optimisation of a person’s medicines is “important to ensure a person is taking their medicines as intended” in order to “obtain the best possible outcomes from their medicines” through “a person-centred approach” [20, 50]. Here the emphasis is on the patient perspective as one of four guiding principles where the patient’s beliefs, values and circumstances are to be considered when making treatment choices as guided by ‘a person-centred approach’ [20, 51]. Such beliefs, values and circumstances can only be recognised through enabling the patient to share their narrative in a genuine non-judgemental manner under the premise of a mutually respectful partnership between the patient and their clinician as with person-centred care [41, 43, 49]. Those participants who referred to having adapted their lifestyle or behaviour in order to accommodate their medicines are not unique as discussed by Gamble *et al* under the theme “impact of lifestyle” in medicine-taking behaviour in patients with difficult asthma [46] where adapted lifestyle is discussed to further effect interaction with family members such as the participant who described loss of a social circle due to their no longer being able to drink alcohol. In contrast, other participants described new friends that had been made through the formation of newer habits as recommended such as going to the gym on a regular basis.

The clinical consultation where these ‘positive’ habits can be honestly and openly discussed should be equally open to discuss those adaptions that don’t fit the patient and their lifestyle. The patient should not have to censor what they disclose to their clinician due to a feeling of guilt or perceived disapproval [53]. If a clinician can assume that medicines are safe to prescribe and use, then a patient should be equally able to discuss openly and honestly their thoughts around medicines as the final action of use lies ultimately with them.

### Methodological considerations

As in line with qualitative and phenomenological hermeneutical research, our findings cannot be (nor is it intended to be) statistically generalised to the larger population. However, regarding transferability, our findings contribute to a deeper understanding of the meanings of medicine taking among people diagnosed with CHF or COPD which can be applied to further develop working methods when prescribing or following up medicine taking within the Swedish primary healthcare system. Studies in different care settings and geographical contexts are recommended to further explicate these findings. Trustworthiness was strengthened by an on-going interpretative process in collaboration between the authors. Credibility was also considered and ensured by the interpretation being based on the original source, the interview text, which is illustrated by citations exemplified from the subthemes and themes. Self-reflexivity was engaged in where the interviewer was aware of their background as a pharmacist and hence the challenge of coming from a typically positivist biomedical background and moving into a constructivist sociological arena [54]. When asked directly by participants if they were a pharmacist, the interviewer was mindful to assure these participants that their responses were not being judged, but that they were more interested in lived experiences of medicines rather than which medicines the participants were prescribed. Further, the lead researcher discussed their concern of looking at the text through a pharmacist lens and how to minimise this with the other researchers throughout the analysis process. However, in phenomenological hermeneutical research, researchers are a part of the research process and hence the findings are to be interpreted within this awareness that this may both limit the findings but also enrich them [54]. Nonetheless, our findings provide novel findings pertaining to patients’ lived experiences of medicines that can be used to inform future research. The results may be useful in informing conceptual and theoretical generalisations.

## Conclusion

In conclusion, the results from this study indicate the need for a co-creation of a treatment plan in partnership between patients and healthcare providers when prescribing medicines. This research can be seen as a call to action for researchers who focus on improving medicine-taking to recognise the importance of the patients’ lived experiences of medicine-taking and how this may impact on their actual medicine-taking.

## Supporting information

S1 File Interview guide. English translation of the interview guide.
(PDF)
