# Supplementary material for: Why do patients struggle with their medicines?—A phenomenological hermeneutical study of how patients experience medicines in their everyday lives
Source: PLoS One. 2021 Aug 6;16(8):e0255478. doi: 10.1371/journal.pone.0255478 (PMC8345846; doi:10.1371/journal.pone.0255478)
Supplement: S1 File — English translation of the interview guide. (PDF) [file pone.0255478.s001.pdf]

Interview guide

- *What do you think about when you think about your medicines?*

Support questions/phrases:

- Tell me about your medicines…
- What is your experience when it comes to medicines?
- Are you satisfied with your medicines?
  - Do you have any problems with your medicines? Now or in the past?
- Have you discussed this (before) with anyone within healthcare,
  - Your doctor or at the pharmacy?
- What do you want when it comes to your medicines?
  - Support?
  - From whom?
- What does […......] mean for you in your everyday life?

Opening phrases:

I really only have one main question to start with and then I will ask further questions based on what you say

Here, I have different prompts….

Would you like to tell me about…?

I am interested to hear what you think about ….
